# Homozygous Familial Hypercholesterolemia: Case Series and Review of the Literature

**DOI:** 10.1155/2011/154908

**Published:** 2012-01-11

**Authors:** Carlos H. Palacio, Theresa R. Harring, N. Thao T. Nguyen, John A. Goss, Christine A. O'Mahony

**Affiliations:** ^1^Michael E. DeBakey Department of Surgery, Baylor College of Medicine, One Baylor Plaza, Suite No. 404D, Houston, TX 77030, USA; ^2^BCM Liver, Kidney, and Pancreas Center, Division of Abdominal Transplantation, Michael E. DeBakey Department of Surgery, Baylor College of Medicine, 1709 Dryden Street, Suite No. 1500, Houston, TX 77030, USA

## Abstract

*Introduction*. Familial hypercholesterolemia (FH) is caused by nonfunctioning low-density lipoprotein (LDL) receptors, resulting in high serum cholesterol. Two types of FH are described: the heterozygous form is diagnosed in adults and responds well to medical therapy; the homozygous form is rare, diagnosed in children, and often requires multiple treatments to prevent complications. Cholesterol accumulation in tissues produces common clinical manifestations including cutaneous xanthomas, coronary artery disease, and aortic stenosis. Treatment options consist of lifestyle modifications, lipid-lowering medications, LDL aphaeresis, and orthotopic liver transplantation (OLT). *Case Presentation*. Two patients with FH presented at young ages due to characteristic cutaneous xanthomas. The patients underwent cardiac testing that revealed atherosclerotic changes. The patients received maximal medical therapy, but only experienced a small decrease in serum cholesterol and LDL levels. After several years of medical treatment without improvement of symptoms, the patients were listed for OLT. The transplantations were successful, and only one patient had a postoperative complication of acute rejection, treated successfully. Currently, both patients are doing well with regression of the cutaneous xanthomas and atherosclerotic changes. *Conclusion*. OLT is a safe and effective option for patients with homozygous FH refractory to maximal medical therapy and may represent the optimal treatment for these patients.

## 1. Introduction

Familial Hypercholesterolemia (FH) is an autosomal dominant genetic disorder due to mutations in the low-density lipoprotein (LDL) receptor gene located on chromosome 19 [1]. There are two types of familial hypercholesterolemia: the heterozygous form in which the patient has one normal allele and one mutated allele is the most common form with an incidence of 1 out of 500, whereas the homozygous form in which the patient has two mutated alleles, considered an autosomal codominant disorder, is rare with an incidence of approximately one in a million [2]. Patients with heterozygous FH are usually diagnosed as adults and often times respond well to medical therapy. On the other hand, patients with homozygous FH are often diagnosed early in childhood, do not respond well to medical therapy, and can progress rapidly to premature coronary artery disease. 

 Low-density lipoproteins (LDLs) and the metabolism of cholesterol are closely entwined, as first theorized in 1973 by Myant [3]. LDL receptors present in the liver clear LDL micelles containing cholesterol from plasma. If these micelles are unable to be cleared, cholesterol levels rise resulting in atherosclerotic changes in arteries. Furthermore, patients may develop accumulation of cholesterol in other parts of the body leading to the development of cutaneous xanthomas, which are most commonly located in the elbows, hands, knees, and Achilles tendon [1]. Several types of cutaneous xanthomas are recognized and associated with FH including xanthelasma, xanthoma tendineum, and xanthoma tuberosum [4]. Xanthelasmas are yellow-orange slightly raised collections of cholesterol underneath the skin found usually around the eyes or on the eyelids [4]. Xanthoma tuberosum are yellow nodules located on the elbows and knees [4]. They can either be a confluence of eruptive xanthomas or be isolated lesions [4]. Xanthoma tendineum or tendinous xanthoma is subcutaneous tumors in tendons that move with extension [4]. Other types of xanthomas, such as eruptive xanthomas, xanthoma planum, palmar xanthomas, and tuberous xanthomas, are not usually associated with FH [4]. The diagnosis of homozygous FH is based on a family history of elevated cholesterol characterized by cholesterol levels greater than 1000 mg/dL despite maximum medical treatment and the development of xanthomas and atherosclerotic cardiac lesions such as aortic stenosis [2].

Beyond genetic counseling for patients and the families of patients diagnosed with FH, treatment options revolve around decreasing serum cholesterol levels and increasing cholesterol removal. Therapies available include lifestyle modifications focusing on a reduced-fat and reduced-cholesterol diet, pharmacologic treatment affecting cholesterol absorption and metabolism, and LDL aphaeresis to remove LDL from the serum to decrease the levels [1]. Additionally, since first described in 1983 by Starzl et al. OLT has been considered a cure for patients with FH [5]. Since OLT addresses the underlying deficiency, the absence of properly functioning LDL-receptors in the liver, patients undergoing OLT have fast and long-lasting resolution of their hypercholesterolemia. Moreover, these patients may have resolution of atherosclerotic changes and cutaneous xanthomas. The following report describes the cases of two pediatric patients that presented to our center who were subsequently diagnosed with homozygous FH and underwent OLT.

## 2. Case  1

The patient is a 4-year-old African American male who presented with cutaneous xanthomas on the extensor surfaces of his arms, knees, elbows, and Achilles tendon. The patient had these xanthomas since he was 1 year old. The patient was found to have serum cholesterol level greater than 1000 mg/dL and triglyceride level of 170 mg/dL. The patient was started on low-fat and low-cholesterol diet and began medication treatment with atorvastatin and ezetimibe. Despite maximal medical therapy and nutritional modifications, the patient's cholesterol levels still ranged between 500 mg/dL and 600 mg/dL. The patient's family history was remarkable in that both parents had high cholesterol levels and the father had arcus senilis corneae. Due to the patient's clinical history and symptoms and the symptoms present in his immediate family, the patient was diagnosed with homozygous FH. A cardiac evaluation with a magnetic resonance angiogram (MRA) of the heart revealed mild supravalvular aortic narrowing, distal transverse arch, and proximal descending aorta narrowing and a luminal irregularity of descending aorta. The patient remained on maximal medical therapy and dietary modifications, and after three years, he continued to develop new cutaneous xanthomas around his ankles and at the base of his fingers. His cholesterol levels persisted in the range between 500 mg/dL and 600 mg/dL, and he had no regression of his cardiac vascular lesions on MRA. Due to the failure of medical therapy to control his symptoms, the patient was listed for OLT.

The patient successfully underwent deceased-donor OLT. There were no intraoperative or postoperative complications. The patient's immunosuppression regimen consisted of tacrolimus, mycophenolate, and prednisone. The patient's steroids were tapered postoperatively per our transplant center protocol. Atorvastatin was also continued post-operatively. Six months after OLT, the patient's xanthomas had regressed substantially and were much softer. The patient had one postoperative complication 2 years after OLT consisting of an episode of acute rejection, which was successfully treated with steroids. MRAs of the heart were performed annually to ascertain regression of atherosclerotic changes. The MRA 3 years after OLT continued to showed supravalvular aortic narrowing and diffuse, mild narrowing of the distal aortic arch with minimal improvement in the luminal caliber but no change in wall thickness severity. Of note, there was significant improvement in the degree of aortic wall thickness compared to the initial MRA. Currently, the patient is doing well over 3 years after transplant, and his cutaneous xanthomas have completely resolved. The patient has been maintained on tacrolimus and atorvastatin. His most recent cardiac testing including stress test, echocardiogram, and electrocardiography is normal. At his last followup, his serum cholesterol level is 152 mg/dL and LDL level is 75 mg/dL.

## 3. Case  2

**Figure 1:**
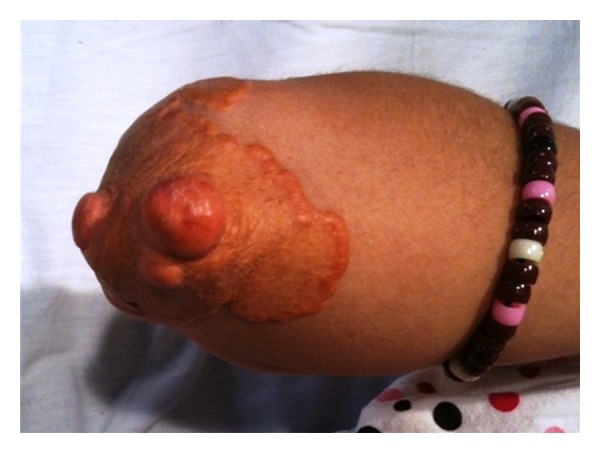
Cutaneous xanthomas on the left elbow of patient no. 2.

**Figure 2:**
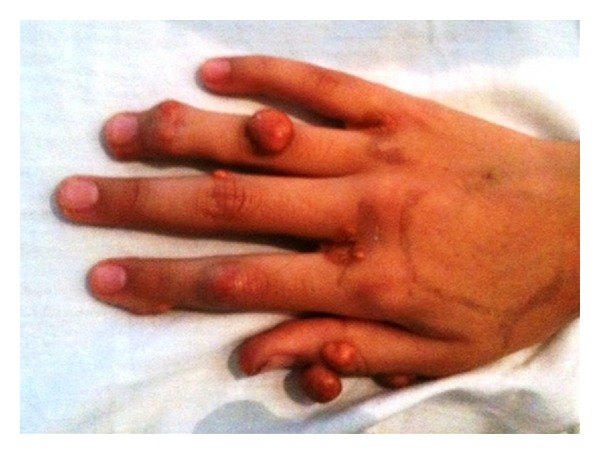
Cutaneous xanthomas on the hand of patient no. 2.

**Figure 3:**
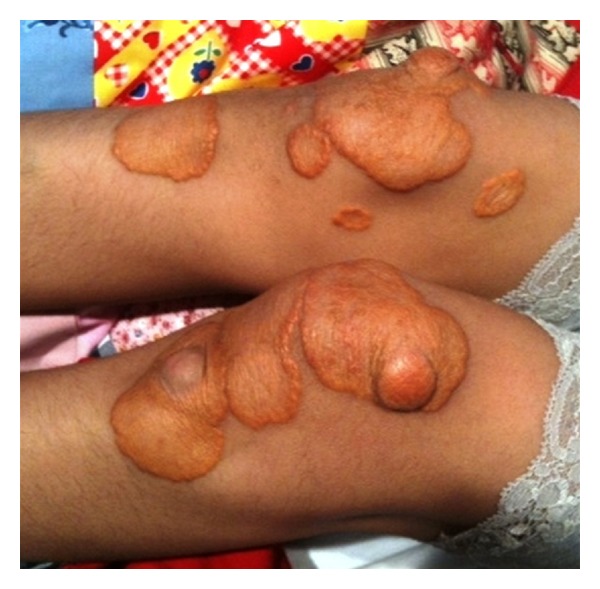
Cutaneous xanthomas on the knees of patient no. 2.

The patient is a Hispanic female diagnosed with homozygous FH at the age of 3 years old. Prior to diagnosis, she had developed multiple cutaneous xanthomas on her hands, feet, elbows, and knees (Figures 1, 2, and 3). Furthermore, the patient complained of chest pain during exertion. Prior to treatment, serum cholesterol ranged between 500 mg/dL and 900 mg/dL. When the patient was 3 years old, she was started on rosuvastatin and ezetimibe. The patient's cholesterol level did not improve; serum cholesterol remained between 600 mg/dL and 700 mg/dL, and serum LDL level was 358 mg/dL. Genetic mutational analysis confirmed the diagnosis of homozygous FH and demonstrated a LDL-receptor genetic mutation with a DNA base change from adenine to guanine. The patient's only family history was a cousin who also had been diagnosed with homozygous FH and underwent OLT.

The patient's cardiac stress test showed no ischemic changes and echocardiogram demonstrated a normal aortic valve with only a trace amount of aortic regurgitation and mild mitral regurgitation, without aortic stenosis. Bilateral carotid artery ultrasound revealed intimal thickening and noncalcified plaques. Computed tomography angiography demonstrated moderate calcification of the aortic valve annulus; calcium-score screening of the right, left main, left anterior descending, and left circumflex coronary arteries was normal. Additionally, cardiac catheterization showed normal coronary arteries without evidence of stenosis or calcification, but also showed mild aortic supravalvular narrowing. The patient was started on LDL aphaeresis due to the exertional angina.

Due to the severity of her symptoms and the failure of medical management, the patient was listed for and then underwent OLT at the age of 11 years old. The patient had no intraoperative or postoperative complications and was discharged home one week later. The patient's immunosuppression consisted of tacrolimus and prednisolone. The steroids were tapered post-operatively. Her serum cholesterol level and LDL level at the time of discharge were 192 mg/dL and 141 mg/dL, respectively. The patient remains free from exertional angina and her serum cholesterol and LDL levels remain in the normal range since her last followup at 6 months after OLT.

## 4. Discussion

In 1939, FH was first described by Muller [6]. Over thirty years later, Myant first documented his theory that increased serum cholesterol was due to an increase in LDL particles [3]. Soon thereafter, Goldstein et al. recognized that FH was a genetically determined defect within hepatocytes [7]. Specifically, he found that the LDL-receptor precursor was not transported to the cell surface in patients with FH; therefore, the LDL-receptors were incapable to bind LDL, resulting in an extraordinary elevation in serum LDL [7]. There are two types of FH that have been described: patients with heterozygous FH usually present as adults and require only medical therapy, but patients with homozygous FH typically present as young children and often have such severe disease that they require LDL aphaeresis or OLT to prevent premature coronary artery disease. Patients with homozygous FH usually present with cholesterol levels as high as or over 1000 mg/dL [1]. The accumulation of cholesterol occurring from birth produces several clinical manifestations, including cutaneous xanthomas, and cardiovascular complications, such as aortic valve disease. The combination of elevated serum cholesterol and LDL levels can lead to early-onset, accelerated atherosclerosis and premature coronary death, usually before the patient turns 30 years old [7]. Genetic counseling for the patient and for the family of patients diagnosed with FH is essential to allow early recognition of other affected individuals and to assess further risk of unaffected individuals.

Cutaneous xanthomas are yellow, elevated lesions, and certain types are found exclusively in patients with FH. These lesions usually develop within the first 4 years of life and oftentimes are the first clue for diagnosis [8]. Cutaneous xanthomas are typically located on the feet, hands, elbows, Achilles tendons, and on subcutaneous and periosteal tissue. Both patients presented had cutaneous xanthomas leading towards the diagnosis of homozygous FH. Other clinical manifestations of FH include xanthomas located in the tongue and buccal mucosa, and arcus senilis corneae, a white-gray opaque ring around the iris [8]. Interestingly, the father of the first patient was found on physical examination to have arcus sinilis corneae.

Another important manifestation of cholesterol deposition includes cardiovascular complications. These cardiac complications can lead to coronary artery bypass grafting in the second decade of life [1], and despite this, patients still may die at a younger age [9]. Additionally, increased serum cholesterol can result in aortic valve stenosis. Deposition of cholesterol and subsequent fibrosis in the aortic valve leads to aortic stenosis [10]. In patients with homozygous FH, the most common cause of morbidity and mortality is due to aortic stenosis [1]. The first patient presented above was found to have supravalvular aortic stenosis diagnosed as part of the cardiac testing. Even 3 years after OLT, the aortic stenosis remained stable without significant changes, underscoring the need for careful followup and screening in these patients.

The management of patients with homozygous FH represents a medical challenge. If left untreated, and sometimes even despite maximal medical therapy, these patients progress rapidly to atherosclerotic changes leading to aortic stenosis and coronary artery disease. The atherosclerotic changes can result in premature myocardial infarction, and untreated patients usually die from coronary artery disease before they turn 20 years old [8]. The noninvasive treatments available to patients with FH include dietary modifications to include a reduced-fat, reduced-cholesterol diet and medical therapy including bile acid sequestrants and HMG-CoA reductase inhibitors or statins. Most patients with homozygous FH actually begin treatment with lipid-lowering medications during the first year of life [10]; unfortunately, these are only partially effective in decreasing cholesterol and LDL levels [1]. Both of the patients presented above were started initially on diet modifications and medical therapy; however, neither patient had a significant decrease in serum cholesterol or LDL levels. Of note, despite treatment with lipid-lowering agents for 3 years, the first patient continued to develop cutaneous xanthomas but, more interestingly, serum cholesterol, LDL, and triglyceride levels decreased by approximately 20%.

Another treatment option for patients with homozygous FH includes LDL aphaeresis. LDL aphaeresis was initially thought to be unsuccessful in the management of homozygous FH; but it actually has been shown to slow progression of coronary atherosclerosis and prolong survival [10]. Although LDL levels can be decreased by 40% to 60% per session, the result is temporary. Higher serum levels return after a median of 3 to 4 weeks in patients with homozygous FH, consequently, regular sessions are required to maintain lower LDL levels [1]. Currently, LDL aphaeresis is recommended at weekly or biweekly intervals with concurrent administration of maximal doses of lipid-lowering agents [10]. Despite the significant decrease in serum LDL levels after treatment, LDL aphaeresis delays but does not prevent the development of atherosclerosis [2, 3]. The second patient presented had one session of LDL aphaeresis due to the timing of OLT.

 The last treatment modality available to patients with homozygous FH is OLT, and it represents the most definitive treatment as it treats the underlying cause of elevated cholesterol: the absence of LDL receptors on hepatocytes. Starzl et al. performed the first OLT for homozygous FH in a 6-year-old patient in 1983 [5]. One year later, Bilheimer et al. demonstrated that the liver has the highest levels of LDL-receptors, proving that a new liver provides a new source of these receptors and therefore decreases cholesterol level levels to normal ranges [11]. Since then, OLT has been recognized as an effective method to definitively decrease cholesterol levels in patients with homozygous FH. Although there are risks inherent to any surgical procedure and there are risks associated with life-long immunosuppression, 5-year survival rates after OLT in pediatric patients approaches 90% in the United States [12].

OLT is specifically indicated for patients with homozygous FH that do not otherwise respond to maximal medical therapy and has been proposed as the only cure for homozygous FH [13]. The case reports described attest to the fact that OLT can safely and effectively treat patients with homozygous FH that are refractory to maximal medical therapy. Both patients received trials of maximal medical therapy with only small decreases in serum cholesterol and LDL. The first patient was treated for 3 years prior to OLT, but his cutaneous xanthomas continued to progress. The second patient was treated medically for 8 years prior to OLT, underwent LDL aphaeresis, yet still had progressive exertional angina. After OLT, both patients had significantly decreased serum cholesterol and LDL levels, and their symptoms and atherosclerotic changes diminished substantially.

Based on these excellent outcomes, and the overall excellent outcomes experienced after OLT with pediatric patients, patients with homozygous FH should be considered candidates for OLT as soon as they fail medical therapy. These patients should be transplanted prior to the development of severe coronary artery disease or aortic stenosis, since these are the most common causes of morbidity and mortality in this population. It is important to note, however, that patients with homozygous FH require complementary medical therapy throughout their lives since the liver is the largest, but not only, source of LDL-receptors. With these factors in mind, excellent outcomes with resolution of symptoms can be achieved after OLT in patients with homozygous FH.

## Abbreviation

FH: Familial hypercholesterolemia
LDL: Low-density lipoprotein
OLT: Orthotopic liver transplantation
MRA: Magnetic resonance angiography.
